# A meta-analysis update evaluating the treatment effects of donepezil alone versus donepezil combined with memantine for Alzheimer's disease

**DOI:** 10.1016/j.ibneur.2025.05.016

**Published:** 2025-05-29

**Authors:** Sajjad Hajihosseini, Seyed Amirali Zakavi, Zahra Farrokhi, Mahnaz Amanzadeh, Parham Panahi, Mina Mahram, Nima Eftekhari, Masoud Noroozi, Mohammad Javad Ebrahimi, Alaleh Alizadeh, Pegah Refahi, Melika Arab Bafrani, Maral Moafi, Niloofar Deravi

**Affiliations:** a Student Research Committee, Tehran University of Medical Sciences, Tehran, Iran; b Students Research Committee, school of medicine, Ardabil University of Medical Sciences, Ardabil, Iran; c Shahid Beheshti University of Medical Sciences, Tehran, Iran; d School of Medicine, Birjand University of Medical Sciences, Birjand, Iran; e Universal Scientific Education and Research Network (USERN), Tehran, Iran; f Student Research Committee, Faculty of Medicine, Hormozgan University of Medical Science, Bandar Abbas, Iran; g Qazvin University of Medical Science, Iran; h Department of Biomedical Engineering, Faculty of Engineering, University of Isfahan, Isfahan, Iran; i Cell Biology and Anatomical Sciences, School of Medicine, Shahid Beheshti University of Medical Sciences, Tehran, Iran; j Faculty of Medicine, Mashhad Branch, Islamic Azad University, Mashhad, Iran; k Student Research Committee, Faculty of Medicine, Mashhad University of Medical Sciences, Mashhad, Iran; l Faculty of Medicine, Tehran University of Medical Sciences, Tehran, Iran; m Student Research Committee, School of Medicine, Shahid Beheshti University of Medical Sciences, Tehran, Iran

**Keywords:** Alzheimer’s disease (AD), Donepezil monotherapy, Combination therapy, Memantine, Mini-Mental State Examination (MMSE), Severe Impairment Battery (SIB)

## Abstract

**Background:**

Alzheimer's disease (AD) remains a significant global health problem, with ongoing debates about the most effective treatment approach. While donepezil monotherapy has been traditionally used, recent interest has focused on combining it with memantine. This updated meta-analysis aimed to compare the efficacy of donepezil monotherapy versus its combination with memantine for treating AD.

**Method:**

A literature search was conducted in the PubMed, Scopus, and Google Scholar databases up to February 14, 2024. Randomized controlled trials (RCTs) comparing donepezil monotherapy with donepezil combined with memantine in AD patients were included. The quality of each selected study was assessed using the Joanna Briggs Institute (JBI) risk-of-bias tool. Data on cognitive function, measured using the Mini-Mental State Examination (MMSE) and the Severe Impairment Battery (SIB), were extracted and analyzed using a random-effects model.

**Results:**

A total of four RCTs, including 1930 patients, met the inclusion criteria. Analysis using a forest plot revealed no significant difference in MMSE scores between monotherapy and combination therapy (OR = 0.54, 95 % CI: 0.06–4.60, p > 0.05). However, SIB scores showed a significant improvement with combination therapy (OR = 7.00, 95 % CI: 1.13–43.24, p < 0.05). Both analyses exhibited high heterogeneity (I² = 72 % for MMSE; I² = 89 % for SIB). The funnel plots suggested minor publication bias for the MMSE outcomes, but some asymmetry was observed in the results for SIB.

**Conclusion:**

This meta-analysis suggests that combination therapy with donepezil and memantine significantly benefits patients with severe cognitive impairment, as assessed by the SIB, compared to donepezil monotherapy. However, no significant advantage was observed in MMSE scores. The high heterogeneity among studies highlights the need for cautious interpretation and calls for larger, well-designed randomized controlled trials to further elucidate the comparative efficacy of these two therapeutic approaches in Alzheimer's disease.

## Introduction

Alzheimer's disease (AD), the most prevalent form of dementia, affects millions of elderly individuals globally. It is a severe neurocognitive disorder marked by a gradual deterioration of cognitive functions and memory, encompassing attention, executive functions, learning, speech, perceptual-motor skills, and social cognition. The resulting impairments often restrict independent daily activities (Maier and Barnikol, 2014, James Siberski, 2012). It is the most common form of dementia, comprising over 80 % of dementia cases in individuals of middle and older age (Kumar et al., 2015). In 2024, approximately 50 million individuals globally were diagnosed with dementia, and this figure is forecasted to nearly double every two decades, reaching 152 million by the year 2050. The underlying biological changes in AD may not always correspond to the first noticeable symptoms. Preceding AD, there is a stage known as mild cognitive impairment, which can vary in duration. This stage implies that patients may live for several years with this cognitive impairment before passing away (Albert et al., 2011, Sperling et al., 2011).

Cholinesterase inhibitors (ChEIs) are approved for dementia regardless of severity, typically prescribed for patients with mild to moderate AD (Mini-Mental State Examination, MMSE score around 26–10). On the other hand, memantine is approved for moderate to severe dementia (MMSE score ≤19). Combination therapy (CT) is often employed in Europe and the United States for individuals with moderate to severe AD (MMSE score 19–10), especially as cognitive function declines (Monsch et al., 2013). These inhibitors work by preventing the breakdown of chemical neurotransmitters, potentially slowing disease progression and delaying cognitive and functional decline. However, the use of ChEIs is associated with mild adverse effects, including diarrhea, nausea, or vomiting (Birks, 2006, Loy and Schneider, 2006, Birks et al., 2015).

An alternative pharmaceutical treatment option for AD is the N-methyl-D-aspartate (NMDA) receptor antagonist memantine, which has been shown to potentially slow disease progression. Interestingly, memantine's side effects are reported to be comparable to those of a placebo (Matsunaga et al., 2015). In June 2010, the United States Food and Drug Administration (FDA) approved a daily extended-release memantine capsule, providing a more user-friendly treatment schedule compared to the twice-daily immediate-release tablets (Namenda XR, 2010). In December 2014, the FDA gave the green light to a once-daily fixed-dose combination of memantine extended-release and donepezil (trade name Namzaric™), which is suitable for the management of moderate-to-severe AD in individuals who have been stabilized on both memantine and donepezil. Additionally, in July 2016, the FDA broadened the indications for this CT to include patients who have been stabilized solely on donepezil (Namzaric, 2014, Deardorff and Grossberg, 2016). Previous systematic reviews examining CT versus monotherapy often overlooked studies that included mild-to-severe dementia (where memantine is not authorized) or used a specific comparator regimen, such as donepezil. A systematic review published in 2017 investigated a topic similar to ours. However, we decided to conduct the current review because many related studies, such as the study conducted by Kim et al. in 2023, have been published since then, adding more data to the field (Kim et al., 2023a). Moreover, the 2017 review reported a high heterogeneity I^2^ = 87.358. This was another reason to write this review is to see if a new analysis could produce results with lower heterogeneity (Schmidt et al., 2015, Tsoi et al., 2019, Chen et al., 2017).

The aim of this updated meta-analysis is to compare the efficacy of donepezil monotherapy versus its combination with memantine in treating AD, with a focus on cognitive improvement. The analysis seeks to determine whether combination therapy demonstrates superiority over monotherapy in these domains, particularly in patients with moderate-to-severe AD, while also assessing safety and tolerability profiles. This meta-analysis is an updated meta-analysis published in 2017 on the comparison of treatment effects in AD between using donepezil as monotherapy versus combining it with memantine.

## Materials and methods

### Study Design

In this systematic review and meta-analysis, we aim to explore the treatment effects of monotherapy with donepezil versus combination with memantine for AD. Our methodology sticks by the PRISMA guidelines (Preferred Reporting Items for Systematic Reviews and Meta-analyses) (Moher et al., 2009). The research protocol for this review has been registered with PROSPERO, registration number CRD42024536151.

### Search Strategy

An advanced literature search was performed up to February 12, 2024, to recover relevant articles from the following databases: PubMed, Scopus, Web of Science, and Cochrane Library. The search strategy consisted of three main subgroups of keywords and Medical Subject Headings (MeSH). One subgroup consisted of terms related to AD, the other two included terms related to memantine and donepezil, respectively. The subgroups were combined using the 'AND' operator, and no limitations were applied regarding the date, publication type, or language. The search strategy was modified according to the format of the query for each database. To reduce the risk of missing related papers, we screened the reference lists of applicable systematic reviews and added studies that met the inclusion criteria of our study. All steps were separately managed by two reviewers, and any debates were settled through conversation between the reviewers. Details of the search strategy and included studies are provided in Table 1.

**Table 1:** Search strategy for selected databases.

Search engine	Search strategy	Additional filters	Total results
Pubmed	(((donepezil[Title/Abstract]) AND (memantine[Title/Abstract])) OR (donepezil[Title/Abstract])) AND (alzheimer*[Title/Abstract])	February 12, 2024	2994
Scopus	1 donepezil 2donepezil and memantine 3alzheimer’s or alzheimer	February 12, 2024	3429
Google Scholar	Donepezil and memantine and alzheimer’s or alzheimer Alzheimer donepezil Memantine or donepezil	February 14, 2024	823

### Inclusion and Exclusion Criteria: Inclusion criteria

For studies to be considered in this meta-analysis, the following criteria should be met:

#### Inclusion Criteria

• Randomized controlled trials (RCTs) only.
• Patients with a diagnosis of Alzheimer's disease at any stage of the disease.
• Comparing the following:

o Donepezil given as a single agent
o Combination of donepezil and memantine

• Reporting at least one of the following:

o MMSE scores^2^
o Severe Impairment Battery (SIB) scores^3^

• English language studies only.
• Studies that provide sufficient data to calculate an effect size, such as odds ratios or mean differences, and their corresponding confidence intervals.

#### Exclusion Criteria

• Observational studies, case reports, case series, commentaries, editorials, and review articles.
• The study population includes those with non-Alzheimer's dementia or those who had mixed dementia.
• Other cholinesterase inhibitors or memantine monotherapy without a donepezil arm.
• Results that did not report the outcomes of interest, namely MMSE or SIB scores, which are clear outcomes of cognitive function.
• High risk of bias or poor methodological detail.
• Studies presented in the form of conference abstracts, unpublished data, or thesis dissertations.

### Data Extraction and Synthesis

Two independent reviewers assessed each study's title and abstract to determine its eligibility for inclusion in this meta-analysis. Studies that did not meet our criteria were excluded. The full text of the remaining studies was screened, and eligible studies were included in the data extraction process. Data were extracted in four categories: 1) Study features (i.e., authors, location, year, and type of study), 2) Patient characteristics (e.g., disease stage, age, comorbidities), 3) Study design (i.e., number of participants, order and period of sampling), and 4) Outcomes related to AD, donepezil concentration, and memantine concentration. Both reviewers used the critical appraisal checklist for RCT studies developed by the Joanna Briggs Institute (JBI) (https://jbi.global/critical-appraisal-tools). A third author participated in the process in case of discrepancies

### Statistical Analysis

We used STATA 13.1 software, developed by StataCorp LP in College Station, TX, USA, for our data analysis. Results were reported as pooled odds ratios (ORs) with a 95 % confidence interval, visualized in a forest plot. We evaluated heterogeneity among the eligible studies using the I^2^ statistic and applied the random effects model when significant heterogeneity was detected (I^2^ > 50 %) (DerSimonian and Kacker, 2007, Higgins and Thompson, 2002). Furthermore, we conducted a sensitivity analysis by excluding one study at a time and repeating the meta-analysis. This allowed us to ensure the stability of our findings. Finally, to investigate the potential for publication bias, we performed a visual inspection of the funnel plot symmetry (Sterne and Harbord, 2004).

The statistical significance level was established at P < 0.05.

## Results

### Study Selection and Baseline Characteristics

**Fig. 1:**
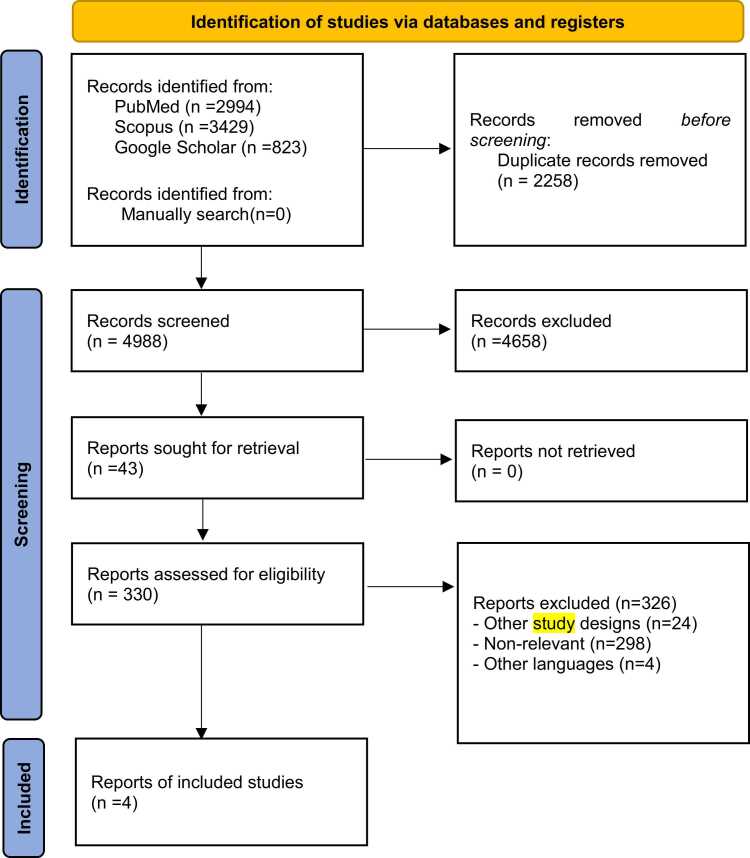
PRISMA flow diagram.

The systematic literature search yielded 7246 articles from PubMed, Google Scholar, and Scopus databases. After removing 2258 duplicates and screening titles and abstracts, 330 studies remained for full-text review. Ultimately, four RCTs met the inclusion criteria and were included in the meta-analysis (Fig. 1).

The included studies, published between 2012 and 2023, collectively enrolled 1930 participants with AD. Of these, 704 patients received CT with donepezil and memantine, while 1100 received donepezil monotherapy. The mean age of participants ranged from 73.6 to 78.8 years. Intervention durations spanned 24–52 weeks, with donepezil dosages ranging from 5 to 23 mg/day and memantine dosages from 5 to 20 mg/day. The studies were conducted in the United States (n = 1), Japan (n = 1), Korea (n = 1), and the United Kingdom (n = 1) (Table 2) (Kim et al., 2023a, Knapp et al., 2017, Doody et al., 2012a, Araki et al., 2014).

**Table 2:** Baseline characteristics of the included studies. Abbreviations: AD = Alzheimer’s Disease D = Donepezil M = Memantine D+M = Donepezil + Memantine USA = United States Of America RCT = Randomized Controlled Trial.

Author/ reference	Year	Country	Study design	Participants	Sex (Fe)	Age	Intervention	Duration	Quality assessment score
Kim et al (Kim et al., 2023a).	2023	Korea	RCT, multicentre, randomized, open-label, and prospective study	Of the 188 participants, 168 completed the ﬁnal research process. D= 95 D+m= 93	DO= 84.2 DM= 45.8	DO= 75.8 ± 6.8 DM= 75.0 ± 8.1	Participants were randomly assigned to one of two treatments: continuation of donepezil (at a dose of 10 mg per day starting in week 1) and continuation of donepezil and initiation of memantine (continuation of donepezil at a dose of 10 mg and memantine at a dose of 5 mg in week 1, with the dose increased in 5 mg increments weekly to a dose of 20 mg from week 4	24 weeks	9 low risk 2 unclear 2 high risk
Knapp et al (Knapp et al., 2017).	2016	United Kingdom	RCT, multicentre, double-blind, placebo-controlled, factorial clinical trial	291 patients with moderate/severe AD D= 73 M= 74 D+m= 72 Placebo= 72	Donepezil alone= 70 % Placebo= 64 % Memantine alone= 61 % Donepezil plus memantine= 67 %	Donepezil alone= 77.2 Placebo= 77.7 Memantine alone= 76.2 Donepezil plus memantine= 77.5	(i) continue donepezil 10 mg per day with placebo memantine; (ii) discontinue donepezil (following 4 weeks donepezil 5 mg) with placebo memantine; (iii) discontinue donepezil and initiate memantine 20 mg per day; and (iv) continue donepezil 10 mg per day and initiate memantine 20 mg per day.	52 weeks	13 low risk
Araki et al (Araki et al., 2014).	2014	Japan	RCT	37 patients with moderate-to-severe AD D= 18 D+m= 19	51.35 %	Mean age 78.8 ± 7.7year(	A memantine combination donepezil group (combination group, n = 19, 77.9 ± 9.8 years) and a non-memantine combination donepezil group (control group, n = 18, 79.8 ± 4.6years)	24 weeks	9 low risk 4 unclear
Doody et al (Doody et al., 2012a).	2012	USA	RCT double-blind multinational trial	1434 moderate-to-severe Alzheimer’s disease Memantine[+ ] subgroup (n = 520) Memantine[-] subgroup (n = 914)	Memantine[+ ] subgroup= 320 (61.5) Memantine[-] subgroup= 581 (63.6)	Memantine[+ ] subgroup= 73.6 (8.63) Memantine[-] subgroup= 74.0 (8.48)	Patients were randomized to donepezil doses (23 vs. 10 mg/day) and stratified by concomitant memantine use (yes or no).	24 weeks	12 low risk 1 unclear

### Quality Assessment

True randomization methods were used in all four studies to generate comparable baseline characteristics across treatment groups. The trial by Knapp et al. (2017) demonstrated the highest methodological quality, with robust methods for allocation concealment and double-blinding (Knapp et al., 2017).

Blinding procedures varied across the studies: a double-blind method was carried out by Doody et al. (2012) and Knapp et al. (2017), increasing the validity of the results (Knapp et al., 2017, Doody et al., 2012a). The design of an open-label study was performed by Kim et al. (2023), which increased the risk of various biases, such as the placebo effect and observer bias (Kim et al., 2023a). Araki et al. (2014) did not provide any information about blinding, and this issue is ambiguous (Araki et al., 2014). All studies uniformly used validated outcome measures, such as MMSE and SIB, to ensure that the assessment of cognitive function was done reliably. The statistical analyses used were appropriate for all trials, with intention-to-treat principles applied to maintain the integrity of randomization. Generally, follow-up was well reported, although reasons for participant dropout were documented. However, the completeness of follow-up varied, as higher attrition rates were seen in some studies than in others.

The included studies, in general, had moderate to high methodological quality; the most robust design came from Knapp et al. (2017) in the trial (Knapp et al., 2017). For Kim et al., 2023a, Kim et al., 2023b, there was an open-label design, while Araki et al.'s trial (2014) presented unclear details regarding blinding (Kim et al., 2023a, Araki et al., 2014). These quality ratings were therefore considered in interpreting the results of the meta-analyses to ensure appropriate weighting of evidence.

### Quantitative data synthesis

#### Forest plot analysis

**Fig. 2:**
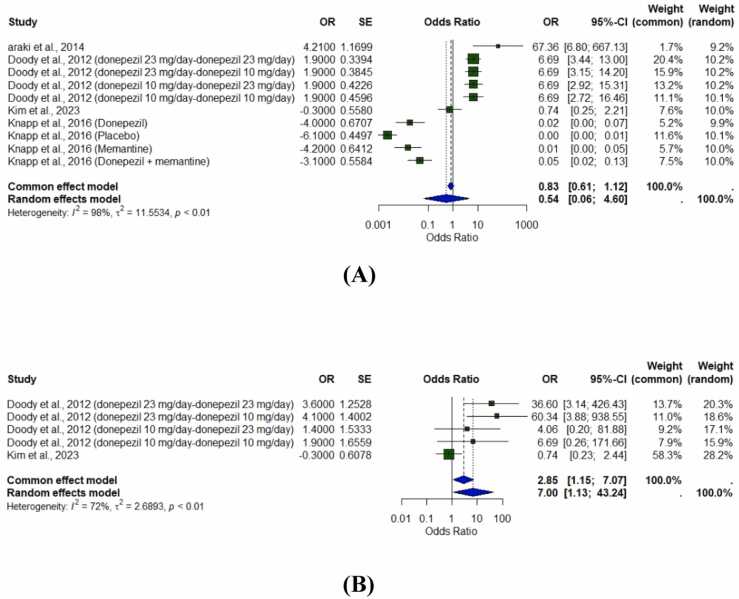
Forest plots of the included studies. (A): Forest plot for Mini-Mental State Examination (MMSE) scores and (B): Forest plot for Severe Impairment Battery (SIB) scores. Abbreviation: CI, confidence interval.

The forest plots for the MMSE and SIB showed different patterns. For MMSE (Fig. 2A), the summary estimated using the common effects model was an OR of 0.83 (95 % CI: 0.61–1.12), and using the random effects model was an OR of 0.54 (95 % CI: 0.06–4.60). Since the confidence intervals crossed 1, there was no significant difference between treatments. We observed high heterogeneity with 72 % I² (p < 0.01), indicating that the results varied significantly among studies.

For SIB (Fig. 2B), the effects were larger: the OR was 2.85 (95 % CI: 1.15, 7.07) using the common effects model and 7.00 (95 % CI: 1.13, 43.24) using the random effects model. This further analysis demonstrated similarly high heterogeneity, with I² of 89 % (p < 0.01), indicating substantial variability in treatment effects.

### Cognitive outcomes

The forest plots for MMSE and SIB, which represented cognitive outcomes, showed divergent results. The forest plot for MMSE (Fig. 2A) indicated no statistically significant difference in scores between the groups receiving monotherapy and CT, as the confidence interval crosses the line of no effect. By contrast, the forest plot for SIB (Fig. 2B) showed much higher scores for CT compared with monotherapy; the overall effect estimate favors CT. Such divergent results suggested that CT may differentially affect the different dimensions of cognitive function in patients with AD.

### Sensitivity analysis

**Fig. 3:**
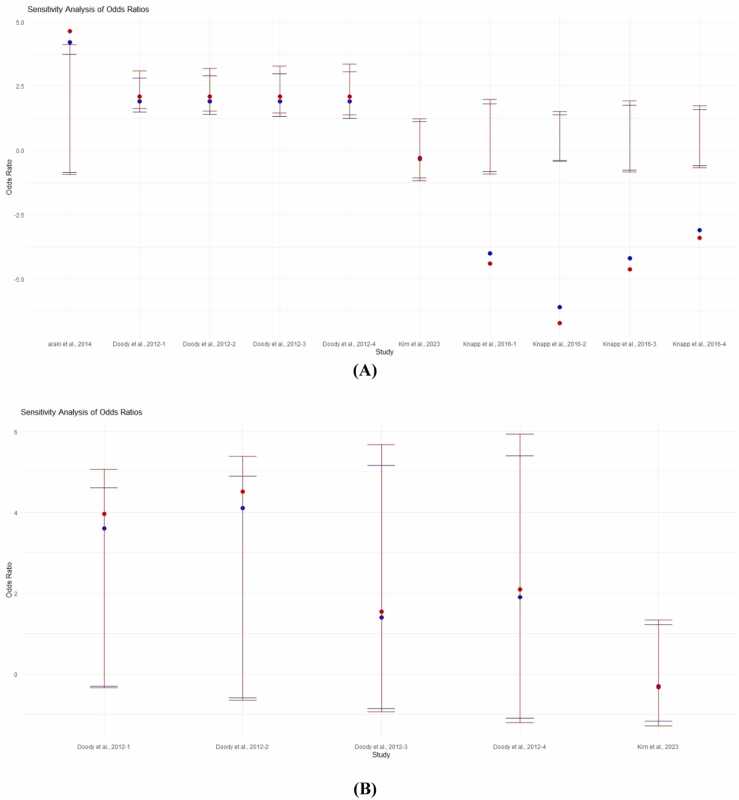
Sensitivity analyses of the included studies. (A): Sensitivity analyses for Mini-Mental State Examination (MMSE) scores and (B): Sensitivity analyses for Severe Impairment Battery (SIB) scores. Abbreviation: OR, odds ratio; CI, confidence interval.

The sensitivity analyses shown in Fig. 3A (MMSE) and 3B (SIB) provided information on the stability of the meta-analysis results. In both figures, the y-axis represents the range of odds ratios for each included study, and points represent individual studies with vertical lines indicating confidence intervals. These analyses also showed that overall results were generally stable and not sensitive to the results of a single research, again reflecting the robustness of conclusions derived from the data.

### Publication bias

**Fig. 4:**
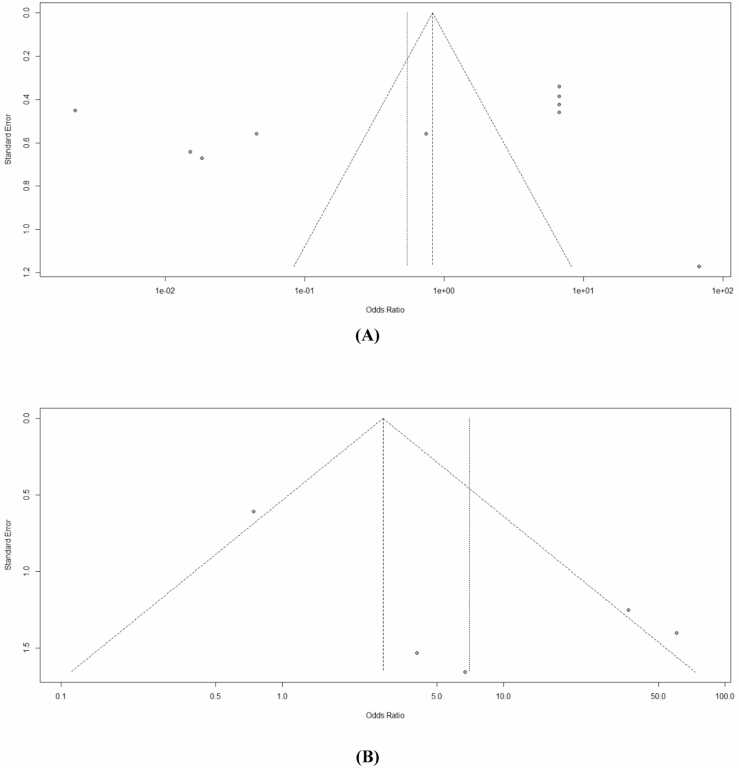
Funnel plots of the included studies. (A): Funnel plot for Mini-Mental State Examination (MMSE) scores and (B): Funnel plot for Severe Impairment Battery (SIB) scores. Abbreviation: SE, standard error; OR, odds ratio.

The funnel plots for MMSE (Fig. 4A) and SIB (Fig. 4B) visually represented the potential for publication bias. For MMSE, the studies were distributed quite symmetrically, indicating minimal publication bias. The SIB funnel plot showed some asymmetry, which could be due to publication bias or the high heterogeneity observed in the analysis. These findings highlight the potential biases underlying these meta-analytic results and caution against overinterpretation, especially for SIB outcomes.

## Discussion

Alzheimer’s treatment targets its core pathological features: acetylcholine depletion, glutamate excitotoxicity, amyloid-β plaques, neurofibrillary tangles, and neuroinflammation. Memantine addresses acetylcholine deficits and glutamate toxicity, countering elevated acetylcholinesterase activity that reduces acetylcholine levels critical for memory processes. By enhancing cholinergic signaling, memantine improves cognitive function. Glutamate binds postsynaptic receptors like NMDA to regulate synaptic transmission, but its dysregulation in Alzheimer’s contributes to neurodegeneration (Morató et al., 2022, Hung and Fu, 2017, Anand et al., 2017). Comparative evaluation of therapeutic strategies reveals memantine's enhanced suitability over ChEIs for advanced AD (Tang et al., 2023). Memantine, as a low-to-moderate affinity uncompetitive NMDA receptor antagonist, blocks receptor overactivation by binding to magnesium-associated sites. This mechanism reduces glutamate-induced neurotoxicity, protecting neurons from damage caused by elevated brain glutamate levels. While aducanumab represents a novel therapeutic pathway, its contentious efficacy data, safety profile debates, and prohibitive costs limit long-term feasibility for most patients (Sánchez-López et al., 2018, Marotta et al., 2020). Despite emerging alternatives, memantine retains critical value in current treatment paradigms due to its established efficacy, tolerability, and accessibility (Tang et al., 2023). Given the pharmacological properties of memantine, it is probable that its benefits would be more evident with prolonged use. However, in some studies, the duration of observation was insufficient to detect any significant improvements in language function (Kim et al., 2023b).

A study suggests that CT with memantine and donepezil could potentially prolong the lives of approximately 303,000 individuals with AD in the United States. In contrast, without treatment or with monotherapy using memantine or donepezil, the life expectancy of these patients would likely be less than five years (Yaghmaei et al., 2024). A 2017 meta-analysis demonstrated the credible efficacy and safety of memantine in the treatment of AD when used as monotherapy or in combination with ChEIs (Kishi et al., 2017). Clinical trials involving patients with AD have indicated that memantine can provide meaningful benefits over placebo in various clinical global measures, including cognition, function, and behavior, while also demonstrating a favorable safety profile and being well tolerated (Parsons et al., 2007, Reisberg et al., 2003, Peskind et al., 2006, Doody et al., 2007, Winblad et al., 2007, Ferris et al., 2009, Tariot et al., 2004). Furthermore, a comprehensive analysis by Atri et al. (2015), combining data from multiple randomized trials, demonstrates that adding memantine to a stable donepezil regimen provides additional clinical benefits and overall clinical status over 24 weeks (Atri et al., 2015). While not formally approved, the memantine-ChEI combination shows promise in advanced Alzheimer’s management, endorsed by clinical guidelines for synergistic symptom relief. Research indicates enhanced cognitive outcomes and global functioning scores compared to single-drug regimens, though patient tolerance varies (Morató et al., 2022, Knorz and Quante, 2022).

The recent panel from the European Federation of Neurological Societies on dementia ultimately endorsed CT as preferable to ChEI monotherapy. This recommendation was deemed weak regarding activities of daily living, cognitive functioning, and overall clinical impression, but was considered strong regarding behavioral outcomes (Schmidt et al., 2015). Nonetheless, the overall clinical advantages of memantine for AD patients remain unclear, particularly with donepezil. Some studies have found that individuals with moderate to severe Alzheimer's who continued treatment with donepezil experienced cognitive improvements that exceeded the minimum clinically important difference, along with significant functional enhancements over 12 months, compared to those treated with memantine (Howard et al., 2012).

Language impairment is one of the most prominent and distressing cognitive deficits associated with AD (Tariot et al., 2004, Blair et al., 2007). Although numerous clinical studies have shown improvements in speech function after memantine treatment, research specifically targeting speech function in AD patients is limited. Furthermore, there are currently no studies available globally that examine the impact of combining donepezil and memantine on language function in patients with moderate to severe stages of AD (Kim et al., 2023b).

The Western Aphasia Battery (WAB)^4^ test revealed a trend towards improvement in language at 24 weeks compared to baseline in both treatment groups. In one study, the donepezil group showed steady improvement, while the combination group initially experienced worsening during the dose-up process, followed by recovery after reaching the full dose. This pattern suggests that memantine may have a positive effect on long-term language function. Compared to the memantine group, the donepezil treatment group showed improvements in cognition (as measured by MMSE and SIB- Short Form) and clinical status (as indicated by Clinical Dementia Rating - Sum of Boxes (CDR-SB), Rosen Modified Hachinski Ischemic Score, Global Deterioration Scale (GDS), and Neuropsychiatric Inventory Questionnaire (NPI-Q)^5^) (Clerici et al., 2012).

An analysis suggests that adding memantine does not significantly affect the cognitive benefits of increasing donepezil from 10 mg/day to 23 mg/day. In the memantine[+ ] and memantine[–] subgroups of this analysis, donepezil 23 mg/day provided statistically significant cognitive benefits over donepezil 10 mg/day, as measured by the SIB. However, on the co-primary global function measure, Clinician’s Interview-Based Impression of Change plus caregiver input (CIBIC-plus)^6^The incremental benefits of donepezil 23 mg/day over donepezil 10 mg/day were not statistically significant, regardless of concomitant memantine use. Similarly, on the secondary measures, including Alzheimer’s Disease Cooperative Study-Activities of Daily Living severe version (ADCS-ADLsev)^7^ and MMSE, donepezil 23 mg/day did not provide any additional benefit over 10 mg/day in either subgroup. Donepezil 23 mg was found to be generally safe and well-tolerated, regardless of whether patients were receiving donepezil alone or in combination with memantine therapy (Doody et al., 2012b).

Donepezil treatment for Alzheimer’s frequently triggers gastrointestinal issues (nausea, vomiting, diarrhea), psychological effects (irritability, anxiety), and slowed heart rate (Deardorff et al., 2015, Campos et al., 2016). Memantine primarily causes digestive discomfort (nausea, diarrhea, constipation) and psychological symptoms like confusion or agitation (Porsteinsson et al., 2008, Raina et al., 2008). Although the incidence of nausea and vomiting was lower and diarrhea was higher in patients taking memantine, the overall rate of gastrointestinal events did not differ between the memantine and non-memantine groups. Most gastrointestinal events occurred within the first month of increasing donepezil from 10 mg/day to 23 mg/day. Subsequently, the incidence of these events among patients receiving donepezil 23 mg/day, with or without memantine, was low and comparable to that observed in patients receiving donepezil 10 mg/day, with or without concomitant memantine (Doody et al., 2012b). Combining both medications does not increase adverse event rates compared to donepezil monotherapy, indicating comparable tolerability between combination and single-drug regimens (Chen et al., 2017). Among 2400 adverse reaction reports linked to donepezil-memantine use, most cases involved women (55 %) and individuals over 65 (79 %). Adverse events typically arose within 19 days of treatment initiation, with most occurring within the first month, though some persisted beyond one year. Reported effects spanned 22 physiological systems, with dizziness and heart rhythm irregularities being frequent, alongside unlisted risks like falls, muscle rigidity, and involuntary muscle spasms. Gender disparities emerged: drowsiness predominated in women, while men more often experienced dystonic posturing. Age-specific patterns showed lethargy as the only common effect across pediatric, adult, and elderly groups (Yang et al., 2024).

Compared to monotherapy with donepezil or memantine, the combination treatment is associated with significantly lower rates of severe clinical deterioration while maintaining good safety and tolerability. Key limitations of CT include reduced physiological adaptability and elevated adverse effects relative to memantine alone. Despite therapeutic potential, evidence gaps regarding long-term safety and optimal dosing necessitate further investigation before widespread clinical adoption (Isaac et al., 2022). The effect sizes generated by this CT are not only statistically significant but also clinically meaningful, underscoring its potential as an effective treatment approach for patients with moderate to severe AD (Atri et al., 2013). However, in the study by Kim et al., 2023a, Kim et al., 2023b, adding memantine to a donepezil regimen did not significantly improve cognitive function or behavioral symptoms in patients with moderate to severe AD. Although both treatment groups showed some initial improvement, these benefits were not sustained over 24 weeks, and the disease continued to progress in both groups. The addition of memantine to donepezil therapy did not result in a statistically significant change in the Korean version of the WAB scores reflecting language function as the primary outcome. Rosen Modified Hachinski Ischemic Score (ROSA) scores revealed improvements in the donepezil-only treatment group. Furthermore, the Neuropsychiatric Inventory (NPI)^8^ measure, as the secondary outcome, also demonstrated symptom amelioration in the CT cohort (Kim et al., 2023b). The study by Howard et al. (2012) found that the effectiveness of donepezil and memantine, as measured by standardized MMSE^9^ and Bristol Activities of Daily Living Scale (BADLS)^10^ scores, was similar regardless of whether the other medication was present. The study did not find any significant advantages to combining donepezil and memantine over using donepezil alone (Howard et al., 2012).

Additionally, some RCTs investigated donepezil monotherapy in patients with mild cognitive impairment (MCI). They demonstrated a reduction in falls in MCI by targeting the motor cognition interface, which has cognitive benefits, and they showed that donepezil is safe for MCI treatments. Moreover, due to its limited benefits and potential side effects, such as diarrhea and insomnia, it is not a recommended treatment. It is recommended that future studies also investigate the effects of monotherapy and CT in this context (Balazs et al., 2021, Salloway et al., 2004, Montero-Odasso et al., 2019, Doody et al., 2010).

A total of 4 RCTs were published between 2012 and 2023, including 1930 participants: 704 in the donepezil and memantine group and 1100 in the donepezil group. This meta-analysis indicates that adding memantine to donepezil increased SIB score but decreased MMSE score compared to the donepezil group; however, between-study heterogeneity was reported as high.

Finally, in this study, the SIB and MMSE indices, which measure cognition, improved compared to baseline in both groups, consistent with the findings of other studies such as Tariot et al. (2004), Schmidt et al. (2015), and Atri et al. (2015). In contrast, the studies by Kim et al., 2023a, Kim et al., 2023b and Howard et al. (2012) found the opposite. In the analysis conducted during this study, the NPI index was not significant in the groups. The funnel plot analysis did not indicate any evidence of publication bias. Due to high heterogeneity among the included studies, further RCTs are needed to support these results.

The strengths are that this meta-analysis has undertaken an extensive search strategy, includes recent studies until 2024, and applies a variety of cognitive and functional outcome measures. Symmetrical funnel plots indicated a low risk of publication bias, increasing confidence in our findings. However, there are several limitations. The high heterogeneity observed across the studies indicated important variability in the treatment effects related to different populations, intervention protocols, or outcome assessments. Moreover, there is a lack of sufficient studies investigating the influence of APOE allelic variations and sex differences in this context. Future RCTs should stratify by APOE genotype and sex to explore differential responses, which may improve treatment strategies. As a result, a comprehensive analysis of these variables was not feasible. Future research is encouraged to explore these aspects of the study. Several studies with limited participants may restrict the generalization of our findings. Further, some studies had a short follow-up period and variable treatment duration, which might have restricted the detection of certain outcomes that required longer observation.

In patients with moderate to severe AD, the combination of memantine and donepezil could be useful in improving cognitive functions, particularly those assessed by the SIB. This will no doubt direct clinical practice, particularly for those patients who do not respond adequately to donepezil alone. However, such a trend was not reflected in the MMSE scores, showing variability of overall results, and therefore, treatment must be highly individualized. Some trials suggest that there is a longer life expectancy of more than five years due to CT, which will significantly alter the management of patients and healthcare planning. Clinicians should consider a balanced benefit from such therapy with the increasing complexity of the medication regimen and additional side effects.

Future studies will have to target some specific areas to strengthen our knowledge regarding CT in AD. Long-term studies with longer follow-ups will be required to establish the long-term efficacy and safety of such treatments, considering the progressive nature of the illness. Subgroup analyses are necessary in determining the patient characteristics predictive of superior responses to CT that allow personalized treatment strategies. The inclusion of complete quality of life assessments in future trials will provide much-needed information concerning the overall effect that CT has on a person and their caregivers. Besides that, the biomarker data would help to present the mode of action behind CT and even the early markers of response to therapy. Strong economic evaluations are needed to truly help form healthcare policy and resource allocation decisions, given the potential of extended life expectancy. This should, in turn, reduce inter-study heterogeneity since, eventually, standardized protocols for treatments and assessments would develop. Hence, more robust meta-analyses will be allowable in the future.

## Conclusion

Memantine can be used effectively in AD. The effectiveness of combining memantine with donepezil versus using donepezil alone is debated. While both combinations can have different effects on various indices (such as the Alzheimer’s Disease Cooperative Study and Clinician’s Interview-Based Impression of Change), these effects can vary significantly.

This study investigated the effects of different donepezil dosages, both with and without memantine, on treatment outcomes. The analysis indicates a significant increase in the score of SIB along with a significant decrease in the score of MMSE. Further RCT studies are needed to confirm these findings.

## CRediT authorship contribution statement

**Sajjad Hajihosseini:** Writing – review & editing, Writing – original draft, Data curation. **Pegah Refahi:** Writing – review & editing. **Alaleh Alizadeh:** Writing – original draft. **Mohammad Javad Ebrahimi:** Writing – original draft. **Masoud Noroozi:** Writing – review & editing, Writing – original draft. **Nima Eftekhari:** Writing – original draft. **Mina Mahram:** Writing – review & editing, Writing – original draft. **Parham Panahi:** Writing – review & editing, Writing – original draft. **Mahnaz Amanzadeh:** Writing – review & editing, Writing – original draft. **Niloofar Deravi:** Validation, Project administration, Conceptualization. **Zahra Farrokhi:** Writing – review & editing, Writing – original draft. **Maral Moafi:** Supervision, Project administration, Methodology. **Seyed Amirali Zakavi:** Writing – original draft, Data curation. **Melika Arab Bafrani:** Software, Formal analysis.

## Ethics approval and consent to participate

Not applicable.

## Compliance with ethical standards

Not applicable.

## Consent for publication

Not applicable.

## Funding

None.

## Declaration of Competing Interest

The authors have no conflicts of interest to declare regarding the study described in this article and preparation of the article.

## Data Availability

Data is available upon request from the corresponding author.

## Footnotes

2 The Mini-Mental State Examination (MMSE) is a widely used test that measures cognitive function. It assesses various cognitive domains, including orientation, memory, attention, language, and visuospatial skills.

3 The Severe Impairment Battery (SIB) is a tool designed to evaluate cognitive function in patients with severe Alzheimer’s disease. It focuses on assessing basic cognitive abilities such as attention, orientation, language, and memory.

## Footnotes

4 Western Aphasia Battery - A comprehensive tool used to assess the linguistic and non-linguistic skills of individuals with aphasia, aiding in the diagnosis and classification of the type and severity of aphasia.

5 These are clinical tools used to assess various aspects of dementia. The CDR-SB quantifies the severity of dementia by summing scores from multiple domains. The Rosen Modified Hachinski Ischemic Score helps differentiate between types of dementia, particularly vascular dementia and Alzheimer’s disease. The GDS stages cognitive decline in dementia patients, and the NPI-Q evaluates neuropsychiatric symptoms such as delusions and depression.

6 A global measure used to assess changes in a patient’s condition based on clinician and caregiver input.

7 The Alzheimer's Disease Cooperative Study-Activities of Daily Living Inventory for Severe Alzheimer's Disease

8 A questionnaire used to assess neuropsychiatric symptoms in individuals with dementia, including delusions, hallucinations, and depression.

9 The Standardized Mini-Mental State Examination [SMMSE, on which scores range from 0 to 30, with higher scores indicating better cognitive function]

10 The Bristol Activities of Daily Living Scale [BADLS, on which scores range from 0 to 60, with higher scores indicating greater impairment]
